# Activation of Pannexin-1 channels causes cell dysfunction and damage in mesangial cells derived from angiotensin II-exposed mice

**DOI:** 10.3389/fcell.2024.1387234

**Published:** 2024-04-09

**Authors:** Claudia M. Lucero, Laura Navarro, Cristián Barros-Osorio, Patricio Cáceres-Conejeros, Juan A. Orellana, Gonzalo I. Gómez

**Affiliations:** ^1^ Institute of Biomedical Sciences, Faculty of Health Sciences, Universidad Autónoma de Chile, Santiago, Chile; ^2^ Departamento de Neurología, Escuela de Medicina and Centro Interdisciplinario de Neurociencias, Facultad de Medicina, Pontificia Universidad Católica de Chile, Santiago, Chile

**Keywords:** panx1, hypertensive nephropathy, intracellular Ca^2+^, ATP, inflammation

## Abstract

Chronic kidney disease (CKD) is a prevalent health concern associated with various pathological conditions, including hypertensive nephropathy. Mesangial cells are crucial in maintaining glomerular function, yet their involvement in CKD pathogenesis remains poorly understood. Recent evidence indicates that overactivation of Pannexin-1 (Panx1) channels could contribute to the pathogenesis and progression of various diseases. Although Panx1 is expressed in the kidney, its contribution to the dysfunction of renal cells during pathological conditions remains to be elucidated. This study aimed to investigate the impact of Panx1 channels on mesangial cell function in the context of hypertensive nephropathy. Using an Ang II-infused mouse model and primary mesangial cell cultures, we demonstrated that *in vivo* exposure to Ang II sensitizes cultured mesangial cells to show increased alterations when they are subjected to subsequent *in vitro* exposure to Ang II. Particularly, mesangial cell cultures treated with Ang II showed elevated activity of Panx1 channels and increased release of ATP. The latter was associated with enhanced basal intracellular Ca^2+^ ([Ca^2+^]_i_) and increased ATP-mediated [Ca^2+^]_i_ responses. These effects were accompanied by increased lipid peroxidation and reduced cell viability. Crucially, all the adverse impacts evoked by Ang II were prevented by the blockade of Panx1 channels, underscoring their critical role in mediating cellular dysfunction in mesangial cells. By elucidating the mechanisms by which Ang II negatively impacts mesangial cell function, this study provides valuable insights into the pathogenesis of renal damage in hypertensive nephropathy.

## 1 Introduction

Chronic kidney disease (CKD) poses a substantial public health challenge, intensified by the increasing prevalence of risk factors and the extension of life expectancy (Eknoyan et al., 2004; WANG et al., 2013; Hoerger et al., 2015). Disturbances in the glomerular and tubulointerstitial compartments play a crucial role in CKD pathogenesis. Certainly, epithelial dysfunction causes injury to the glomeruli, resulting in a reduction in the glomerular filtration rate (GFR) (Nath, 1992; Healy and Brady, 1998; NANGAKU, 2004). In severe cases, this progression may culminate in organ failure and, ultimately, death (Rucker et al., 2011). Numerous elements contribute to the onset of CKD, including diabetes, obesity, and heart disease, with hypertension standing out as one of the most prominent and significant risk factors (Kazancioğlu, 2013). Remarkably, the renin-angiotensin system (RAS) is crucial in blood pressure regulation, and its imbalance can lead to hypertension (Carey and Padia, 2009).

In mammals, the key bioactive component of RAS is angiotensin II (Ang II), which critically regulates blood pressure and fluid balance (Gómez et al., 2019). This peptide hormone is produced through both local and systemic activation of RAS and interacts with two types of membrane G-protein-coupled receptors, known as type I (AT1) and type II (AT2) (da Silva Novaes et al., 2018). The Ang II-mediated activation of AT1 receptors is part of the conventional axe of RAS that leads to vasoconstriction, sympathetic nervous system activation, aldosterone, vasopressin and endothelin secretion, platelet aggregation, cardiac contractility, superoxide formation, vascular smooth muscle cell growth and collagen formation (Carey and Padia, 2009; Sharma et al., 2019 Gómez et al., 2020). Conversely, the actions of Ang II on AT2 receptors belong to the non-conventional or protective arm of RAS (Sharma et al., 2019; Gómez et al., 2020), eliciting the release of kinins (bradykinin, kallikrein), cGMP, and NO levels. The latter facilitates natriuresis, lowering blood pressure along with vasodilatory, anti-inflammatory, and antioxidative stress responses (Danser et al., 1994; van Kats et al., 1998; 2000; Carey and Padia, 2009; Sharma et al., 2019). This intricated balance between the conventional and non-conventional actions of Ang II underscores its complex role in coordinating both physiological and pathological processes.

In the kidney, Ang II actions fundamentally cause renal vasoconstriction via AT1 receptor stimulation, potentially causing inflammation (Sharma et al., 2019; Gómez et al., 2020). This effect is often accompanied by tubulointerstitial ischemia and further glomerular injury, resulting in hyperfunction in the remaining tubules. The latter involves the recruitment of leukocytes, cytotoxicity, and fibrogenesis, negatively impacting the progression of various kidney diseases, including nephropathies, renal artery stenosis, and acute kidney injury (Sharma et al., 2019; Gómez et al., 2020). Mesangial cells play a crucial role in maintaining capillary opening, regulating the GFR, and synthesizing/degrading extracellular matrix proteins. Recent evidence indicates that mesangial cells stimulated with Ang II release inflammatory cytokines and chemokines, initiating extracellular matrix remodeling and eventual fibrosis (Hart and Bakris, 2010; Seccia et al., 2017; Carriazo et al., 2020). However, the molecular mechanisms behind these responses remain to be fully elucidated.

Homeostasis relies heavily on kidney function and kidney cells are intricately tuned to modulate and coordinate their activity. Cell communication is, therefore, vital for normal physiological function. In various organisms, this communication is in part mediated through the release of paracrine molecules through the group of plasma membrane channels denominated large-pore channels (Gajardo-Gómez et al., 2016; Molica et al., 2018). One emblematic protein member of this group was discovered in vertebrates by Panchin and colleagues three decades ago (Panchin et al., 2000). These proteins, called pannexins, include three mammalian members (Panx1–3) (Gajardo-Gómez et al., 2016). Panx1 appears to be the most widespread among the three pannexins, with varying levels of transcripts detected in different tissue types. On the other hand, Panx2 transcripts are primarily found in the brain (Baranova et al., 2004), while Panx3 is most abundant in skin, cartilage, and bone (Celetti et al., 2010; Iwamoto et al., 2010; Ishikawa et al., 2011). Pannexins are tetra-spanning integral membrane proteins with one intracellular loop, two extracellular loops, and both N- and C-terminal tails exposed to the citosol (Yen and Saier, 2007; Penuela et al., 2014). Recently, different independent groups have determined the near-atomic-resolution structure of Panx1 by cryo-electron microscopy, revealing a heptameric channel architecture (Deng et al., 2020; Michalski et al., 2020; Bhat and Sajjad, 2021). Panx1 channels allow the ionic and molecular exchange between the cytoplasm and the extracellular space, contributing to the physiological paracrine and intracellular signaling in different tissues (Barbe et al., 2006; Dahl and Keane, 2012); including the vasculature (Molica et al., 2018). Various stimuli can activate these channels, including the rise of intracellular Ca^2+^ concentration ([Ca^2+^]_i_), membrane depolarization, caspase-mediated C-terminal cleavage, Src kinase-mediated phosphorylation, and interaction with P2X7 receptors (Menzies et al., 2015).

It has been demonstrated that Panx1 channels are a part of the P2X7 receptor complex necessary for ATP release (Silverman et al., 2009). ATP is one of the primary signaling molecules released during the inflammatory response, enhancing inflammasome activation, leukocyte recruitment, and T-cell activation (Rusiecka et al., 2022). Exacerbated ATP release through Panx1 channels promotes the activation of P2X7 receptors, leading to a sustained inflammatory state due to ATP-dependent ATP release. This concept is supported by accumulating evidence linking high Panx1 channel expression and activity to disease onset or progression (Penuela et al., 2014). Additionally, the selective inhibition of Panx1 channels *in vivo* reduces acute inflammatory responses, contributing to better outcomes in various animal models of inflammatory diseases involving different organs, including the kidney and heart (Rusiecka et al., 2022). We recently demonstrated that Ang II increases the activity of Panx1 channels in the mesangial cell line MES-13, resulting in the production of oxidative stress and the release of pro-inflammatory cytokines (Gómez et al., 2018; Lucero et al., 2022a). However, it remains an open question whether *in vivo* administration of Ang II could impact the activity of Panx1 channels in mesangial cells and, if so, how this would affect the function of these cells.

Here, we demonstrate that *in vivo* treatment with Ang II renders mesangial cells susceptible to an elevated Panx1 channel opening upon subsequent *in vitro* treatment with Ang II. Furthermore, this response is accompanied by an increased release of ATP and pro-inflammatory cytokines, coupled with a significant impairment in [Ca^2+^]_i_ dynamics and cell viability. Importantly, the inhibition of Panx1 channels completely prevented the Ang II-induced disturbances in the function and survival of mesangial cells.

## 2 Materials and methods

### 2.1 Reagents and antibodies

Angiotensin II (Ang II) was obtained from Alomone (Jerusalem, Israel), lanthanum (La^3+^) chloride, penicillin (10,000 U) and streptomycin (10 mg/mL), carbenoxolone (CBX), the monoclonal anti-α-tubulin antibody, probenecid (PBC) and malondialdehyde (MDA) were obtained from Sigma‒Aldrich (St. Louis, MO, United States); ^10^panx1 (WRQAAFVDSY, first extracellular loop domain of Panx1) was obtained from GenScript (New Jersey, United States); and A740003, a specific P2X7 receptor blocker, was obtained from Tocris Biosciences (Bristol, United Kingdom). The monoclonal anti-unphosphorylated Panx1 antibody was obtained from Invitrogen (Carlsbad, CA, United States); and anti-mouse and anti-rabbit secondary antibodies conjugated to horseradish peroxidase were obtained from Santa Cruz Biotechnology Inc. (Santa Cruz, CA, United States). HEPES, ATP, NaCl, KCl, CaCl_2_, MgCl_2_ and glucose were obtained from MERCK (Darmstadt, Germany). Fetal bovine serum (FBS) was purchased from HyClone (Logan, UT, United States), whereas trypsin 10X, Hank’s solution, ATP determination kit, FURA-2 AM Dulbecco’s modified Eagle’s medium (DMEM), phosphate-buffered saline (PBS) and ethidium (Etd) bromide (10 mg/mL) were purchased from Thermo Fisher Scientific (Waltham, MA, United States). 4′,6-Diamidino-2-phenylindole (DAPI) was obtained from Abcam (Cambridge, United Kingdom).

### 2.2 Animals

C57BL/6 (U. Chile) mice of 6–8 weeks of age were housed in cages in a temperature-controlled (24°C) and humidity-controlled vivarium under a 12 h light/dark cycle (lights on 8:00 a.m.) with *ad libitum* access to food and water. All procedures were in accordance with institutional and international standards for the humane care and use of laboratory animals (Animal Welfare Assurance Publication A5427-01, Office for Protection from Research Risks, Division of Animal Welfare, NIH (National Institutes of Health), Bethesda, MD, United States). The Bioethical and Biosafety Committee of the Faculty of Biomedical Sciences at Universidad Autónoma de Chile (BE07-20; 26 October 2020) approved the described experimental procedures.

### 2.3 Ang II administration and experimental procedure

We used a well-known model of chronic administration of Ang II that causes hypertension (Giachelli et al., 1994). Briefly, animals were anesthetized with a dose of ketamine/xylazine (25:2.5 mg/kg). A small (1 cm) cut was performed in the mid-scapular region, and an osmotic pump was implanted; subsequently, the wound was sutured. After their recovery, the mice were kept with water and food *ad libitum*. Mice were infused with Ang II (1,000 ng/min) or saline for 6 weeks. Weekly measures were performed to examine various physiological parameters, including blood pressure (see below) (Gómez et al., 2019).

### 2.4 Blood pressure measurements

Systolic and diastolic blood pressure and mean arterial pressure (PAM) were determined once a week in the morning in conscious prewarmed restrained mice by noninvasive plethysmography (NIBP machine, IITC Inc., Woodland Hills, CA, United States) by the tail-cuff method. At least four determinations were made in every session, and the mean of the ten measurements was taken as the blood pressure value.

### 2.5 Renal function measurements

Plasma and urinary creatinine levels were measured by the Jaffé alkaline picrate assay (VALTEK Diagnostica, Santiago, Chile). The urinary protein concentration was determined by Bradford’s method (Bio-Rad protein assay, Kidlington, United Kingdom) (Salas et al., 2003). To determine the U prot/U Crea ratio, the value for urinary protein was divided by the value for urinary creatinine. The levels of blood urea nitrogen (BUN) in serum were determined by appropriate detection kits following the manufacturer’s instructions.

### 2.6 Isolation of primary glomerular mesangial cells

Primary mesangial cells were isolated from mouse glomeruli treated with collagenase (Menè and Stoppacciaro, 2009; Wilson and Stewart, 2012; Salva et al., 2017; Wang et al., 2019). In brief, kidney fragments were minced with a razor blade, and a 100 m nylon sieve was used to collect the glomeruli from the cortex homogenates of mouse kidneys under aseptic conditions. This glomeruli-enriched fraction was collected from underneath the sieve with HBSS. The diluted suspension was poured onto a second 70 m filter and washed with the same solution. The glomeruli and other fragments retained on the filter were transferred into a sterile tube. The glomerular suspension was incubated for digestion with sterile type IV collagenase in DMEM for 1 h at 37°C in an incubator. Then, it was triturated through a 21-gauge needle. Glomerular remnants were washed, and mesangial and endothelial cells were plated onto a six-well plate in a complete medium and incubated at 37°C in a humidified 5% CO_2_ incubator (Menè and Stoppacciaro, 2009; Wilson and Stewart, 2012; Salva et al., 2017; Wang et al., 2019).

### 2.7 Cell treatments

Mesangial cells were treated with 1 µM Ang II for different periods (0, 24, 48 and 72 h) in a 2:1 mixture of DMEM and F-12 tissue culture media supplemented with 100 U/mL penicillin and 100 g/mL streptomycin. Cells were kept at 37°C in 5% CO_2_/95% air at nearly 100% relative humidity. CBX (10 μM), PBC (500 μM) and ^10^panx1 (50 μM) were added 24 h before the end of a 3-day experiment to cell cultures treated with Ang II at time zero.

### 2.8 Dye uptake and time-lapse fluorescence imaging

The activity of Panx1 channels was evaluated by using the Etd uptake method, as previously described (Orellana et al., 2012). In brief, cells at 70% confluence were plated onto glass coverslips and bathed with Locke’s saline solution (in mM: 154 NaCl, 5.4 KCl, 2.3 CaCl_2_, 1.5 MgCl_2_, 5 HEPES, 5 glucose, and pH 7.4) containing 5 μM Etd. Time-lapse images were measured (at regions of interest in different cells) every 30 s for 13 min using a Nikon Eclipse Ti inverted microscope (Tokyo, Japan) and NIS-Elements software. The fluorescence intensity recorded from 25 regions of interest (representing 25 cells per coverslip) was defined as the subtraction (F–F0) between the fluorescence (F) from the respective cell (25 cells per field) and the background fluorescence (F0) measured where no labeled cells were detected. The mean slope of the relationship F-F0 over a given time interval (ΔF/ΔT; F0 remained constant along the recording time) represents the Etd uptake rate. To determine changes in slope measurements, regression lines were fitted to points before and after the various experimental conditions using Excel software, and mean values of slopes were compared using GraphPad Prism software and expressed as AU/min. At least four replicates (four sister coverslips) were measured in each independent experiment (Sáez et al., 2018).

### 2.9 [Ca^2+^]_i_ cell imaging

Mesangial cells plated on glass coverslips were loaded with 5 µM Fura-2-AM in DMEM without serum at 37°C for 45 min and then washed three times in recording solution (in mM): 140 NaCl, 4 KCl, 2 CaCl_2_, 1 MgCl_2_, 5 glucose, and 10 HEPES, pH = 7.4, followed by de-esterification at 37°C for 15 min. The experimental protocol for Ca^2+^ signal imaging involved data acquisition every 10 s for 5 min (emission at 510 and 515 nm, respectively) at 340/380-nm excitation wavelengths (Xenon lamp) using an inverted microscope (Eclipse Ti-U, Nikon). NIS element advanced research software (version 4.0, Nikon) was used for data acquisition and image analysis. The fluorescence intensity recorded in 15 cells involved the determination of pixels assigned to each cell. The average pixel value allocated to each cell was obtained with excitation at each wavelength and corrected for background. The FURA-2 ratio was obtained after dividing the 340-nm by the 380-nm fluorescence image on a pixel-by-pixel basis (R = F_340 nm_/F_380 nm_).

### 2.10 Measurement of ATP

The extracellular amount of ATP was measured using the ATP determination kit according to the protocol provided by its supplier Invitrogen (ATP Determination Kit, A22066). Using the conditioned medium of mesangial cells and applying the kit that includes D-Luciferin and recombinant firefly luciferase, the luminescence produced by luciferin upon binding with ATP was measured using a Tecan Infinite^®^ M200 PRO plate reader spectrometer (Männedorf, Switzerland) and i-control™ software.

### 2.11 Immunofluorescence

Mesangial cells grown on coverslips were fixed at room temperature (RT) with 2% paraformaldehyde for 30 min and then washed three times with PBS. They were incubated three times for 5 min in 0.1 M PBS glycine and then in 0.1% PBS-Triton X-100 containing 10% NGS for 30 min. Cells were incubated with anti-Panx1 polyclonal antibody (1:100) diluted in 0.1% PBS-Triton X-100 with 2% NGS at 4°C overnight. After five rinses in 0.1% PBS-Triton X-100, cells were incubated with goat anti-rabbit IgG Alexa Fluor 555 (1:1,000) at RT for 60 min. After several rinses, coverslips were mounted in Paramount-DAPI fluorescent mounting medium and examined with high-resolution fluorescence microscopy (Leica, Wetzlar, Germany) with a 63X objective.

### 2.12 Western blot assays

Mesangial cell cultures were placed on ice, washed twice with ice-cold PBS (pH 7.4), and harvested by scraping in 80 μL of a solution containing a protease and phosphatase inhibitor cocktail (Thermo Scientific, Pierce, Rockford, IL, United States; cat # 78430). Lysates were centrifuged (25,200X g, Eppendorf Centrifuge 5415C, Hamburg, Germany), and supernatants were collected for Western blot analysis. Protein concentration was determined using Lowry’s method (Lowry et al., 1951). Samples of homogenized cell cultures (50 μg of proteins) under different conditions were resolved by electrophoresis in 10% SDS-polyacrylamide gel, and prestained molecular weight markers were resolved in one lane. Proteins were transferred to a PVDF membrane (pore size: 0.45 μm), which was blocked at RT with Tris pH 7.4, 5% skim milk (w/v) and 1% BSA (w/v). Then, the PVDF membrane was incubated overnight at 4 °C with anti-Panx1 (1:1,000) antibody, followed by incubation with rabbit (1:2,000) for 1 h at RT. Then, the PVDF membrane was stripped and reblotted with the anti-α-tubulin antibody (1:5,000) used as a loading control, following the same procedure described above. After repeated rinses, immunoreactive proteins were detected by using ECL reagents (Pierce Biotechnology, Rockford, IL, United States) according to the manufacturer’s instructions. The bands detected were digitized and subjected to densitometry analysis using ImageJ software (Version 1.50i, NIH, Washington, DC, United States).

### 2.13 RT‒PCR assay

The total RNA of cellular and kidney homogenates was obtained using TRIzol reagent (Ambion). The procedure was established according to the manufacture’s instructions. Aliquots of 2 μg of the whole RNA were transformed into cDNA by using MMLV reverse transcriptase (Fermentas), and the amplicon amount was assessed by PCR amplification (GoTaq flexi DNA Polymerase, Promega). The primers for GADPH were the following: sense ’-ACC​ACA​GTC​CAT​GCC​ATC​AC-’ and antisense ’-TCC​ACC​ACC​CTG​TTG​CTG​TA-’. The primers for Panx1 were the following: sense ’-GTG​GCT​GCA​CAA​GTT​CTT​C-’ and antisense ’-CTC​TGC​CCC​ACA​TTC​TCA​GT-’.

### 2.14 Cell viability

The number of viable cells was quantified using an MTT/PMS reagent-based Cell Titer 96 Aqueous Non-Radioactive Cell Proliferation Assay Kit (Promega) according to the manufacturer’s instructions (Xu et al., 2018). 10 μL of aqueous MTT solution (4 mg/mL) was then added to each well (100 μL), and the mixture was incubated at 37°C for 3 h. The MTT solution was carefully decanted off, and formazan was extracted from the cells with 100 μL of a 4:1 DMSO–EtOH mixture in each well. Color intensity was measured with a 96-well ELISA plate reader at 550 nm, with the reference filter set to 620 nm. All MTT assays were repeated three times.

### 2.15 Enzyme-linked Immunosorbent assay

IL-1β was determined in the extracellular medium under different conditions in mesangial cells. Samples were centrifuged at 14.000 g for 40 min. Supernatants were collected and protein content was assayed using the BCA method. IL-1β levels were determined by sandwich ELISA, according to the manufacturer’s protocol (IL-1β EIA kit, Enzo Life Science, Farmingdale, NY, United States). For the assay, 100 µL of samples were added per ELISA plate well and incubated at 4°C overnight. A calibration curve with recombinant cytokine was included. Detection antibody was incubated at room temperature for 1 h and the reaction developed with avidin–HRP and substrate solution. Absorbance was measured at 450 nm with reference to 570 nm with the microplate reader Synergy HT (Biotek Instruments).

### 2.16 Thiobarbituric acid reactive substances (TBARS) measurement

The amount of TBARS was estimated using the method described by Ramanathan and collaborators (Ramanathan et al., 1994) with slight modifications. Culture medium was mixed with SDS (8% w/v), thiobarbituric acid (0.8% TBA w/v), and acetic acid (20% v/v) and heated for 60 min at 90°C. The material that precipitated was removed by centrifugation, and the absorbance of the supernatant was evaluated at 532 nm. The amount of TBARS was calculated using a calibration curve obtained with malondialdehyde (MDA) as a standard. MDA was obtained from Merck (Darmstadt, Germany).

### 2.17 Data analysis and statistics

The results are expressed as the mean ± standard error of the mean (SEM); *n* refers to the number of independent experiments performed. Statistical analysis was performed using GraphPrad Prism (version 9, GraphPad Software, La Jolla, CA, United States). Normality and equal variances were assessed by the Shapiro‒Wilk normality test and Brown-Forsythe test, respectively. Depending on the nature of the data, they were analyzed with parametric or nonparametric tests. When the data were normal, without unequal variance and nonheteroscedastic, a t-test was used to compare two groups, and in the case of multiple comparisons, one or two-way analysis of variance (ANOVA) was used, followed; in the case of significance, by Bonferroni’s *post hoc* test. When the data were heteroscedastic as well as nonnormal and had unequal variation, nonparametric tests were used, such as the Kruskal‒Wallis test, followed, in the case of significance, by Dunn’s *post hoc* test. Details of the statistical results, together with the n and number of replicates, are included in the figure legends. A probability of *p* < 0.05 was considered statistically significant.

## 3 Results

### 3.1 *In vivo* treatment with Ang II renders mesangial cells susceptible to the activation of Panx1 channels

**FIGURE 1:**
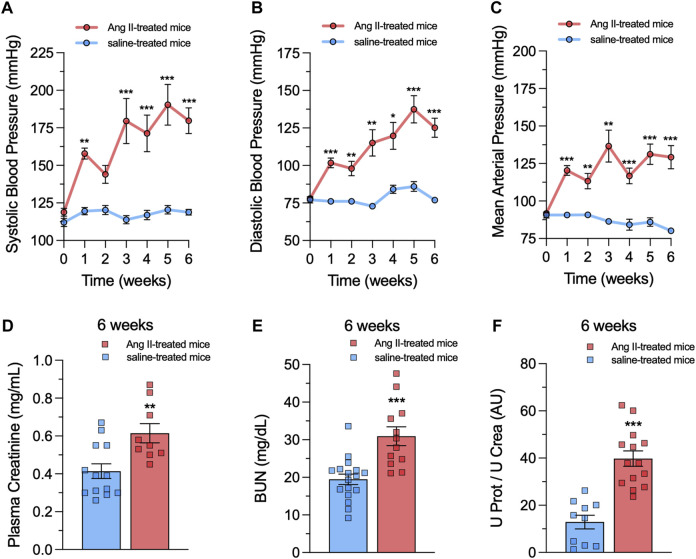
Ang II infusion causes hypertension and renal damage. **(A–C)** Averaged data of systolic blood pressure **(A)**, diastolic blood pressure **(B)** and mean arterial pressure **(C)** over time in mice under control (saline) conditions (blue circles) or infused with Ang II (red circles) for 6 weeks. **(D–F)** Averaged data of plasma creatinine levels **(D)**, blood urea nitrogen (BUN) levels **(E)** and the ratio of urine protein to urine creatinine (U Prot/U Crea, **(F)** in mice under control (saline) conditions (blue bars) or after 6 weeks of infusion with Ang II (red bars). Each bar represents the mean value ± SEM of ≥9 independent experiments. Statistical significance, *p < 0.05, **p < 0.01, ***p < 0.001; Ang II-treated mice compared to saline-treated mice (one-way ANOVA followed by Tukey’s *post hoc* test).

Ang II-mediated activation of AT1 receptors is pivotal in the progressive deterioration of glomerular function, contributing to inflammatory and oxidative damage in renal diseases (Rupérez et al., 2005; Lucero et al., 2022b). Additionally, mesangial cells critically participate in glomerular hypertension and progressive renal disease (da Silva Novaes et al., 2018; Zhao, 2019). We recently found that *in vitro* treatment with Ang II boosts the activity of Panx1 channels in the mesangial cell line MES-13 (Gómez et al., 2018). However, whether *in vivo* treatment with Ang II could modulate the activity of these channels in mesangial cells remains unknown. To explore this question, mice were subjected to a 6-week subcutaneous infusion of either Ang II or saline via osmotic pumps. Previous studies have shown that constant delivery of Ang II under these conditions elicits hypertension in weeks (Menzies et al., 2015; Gómez et al., 2019). Consistently, after 1 week of Ang II infusion, mice showed a substantial rise in systolic blood pressure (Ang II: 163 ± 9 mmHg vs saline: 117 ± 1 mmHg), diastolic blood pressure (Ang II: 111 ± 8 mmHg vs saline: 78 ± 2 mmHg) and mean arterial pressure (Ang II: 120 ± 6 mmHg vs saline: 87 ± 2 mmHg) compared to the control saline group (Figures 1A–C). These responses steadily increased until reaching a plateau around 5–6 weeks of Ang II infusion (Figures 1A–C). The quantification of proteinuria serves as a pivotal diagnostic and treatment monitoring tool in CKD (Clermont et al., 2000). A single sample of urine and plasma, coupled with measurements of creatinine, protein, and blood urea nitrogen (BUN), can provide valuable insights into kidney health and function (Clermont et al., 2000; Methven et al., 2010; Gómez and Velarde, 2018; Gómez et al., 2019). As expected, after 6 weeks of Ang II infusion, mice showed increased levels of plasma creatinine (Ang II: 0.61 ± 0.05 mg/mL vs saline: 0.41 ± 0.04 mg/mL) (Figure 1D), BUN (Ang II: 38.71 ± 5.71 mg/dL vs saline: 19.46 ± 1.39 mg/dL) (Figure 1E) and the ratio of urine protein to urine creatinine (Ang II: 39.75 ± 3.26 AU vs saline: 12.88 ± 2.90 AU) (Figure 1F). These findings collectively indicate that our model of Ang II infusion exacerbates hypertensive conditions and induces renal damage in mice.

**FIGURE 2:**
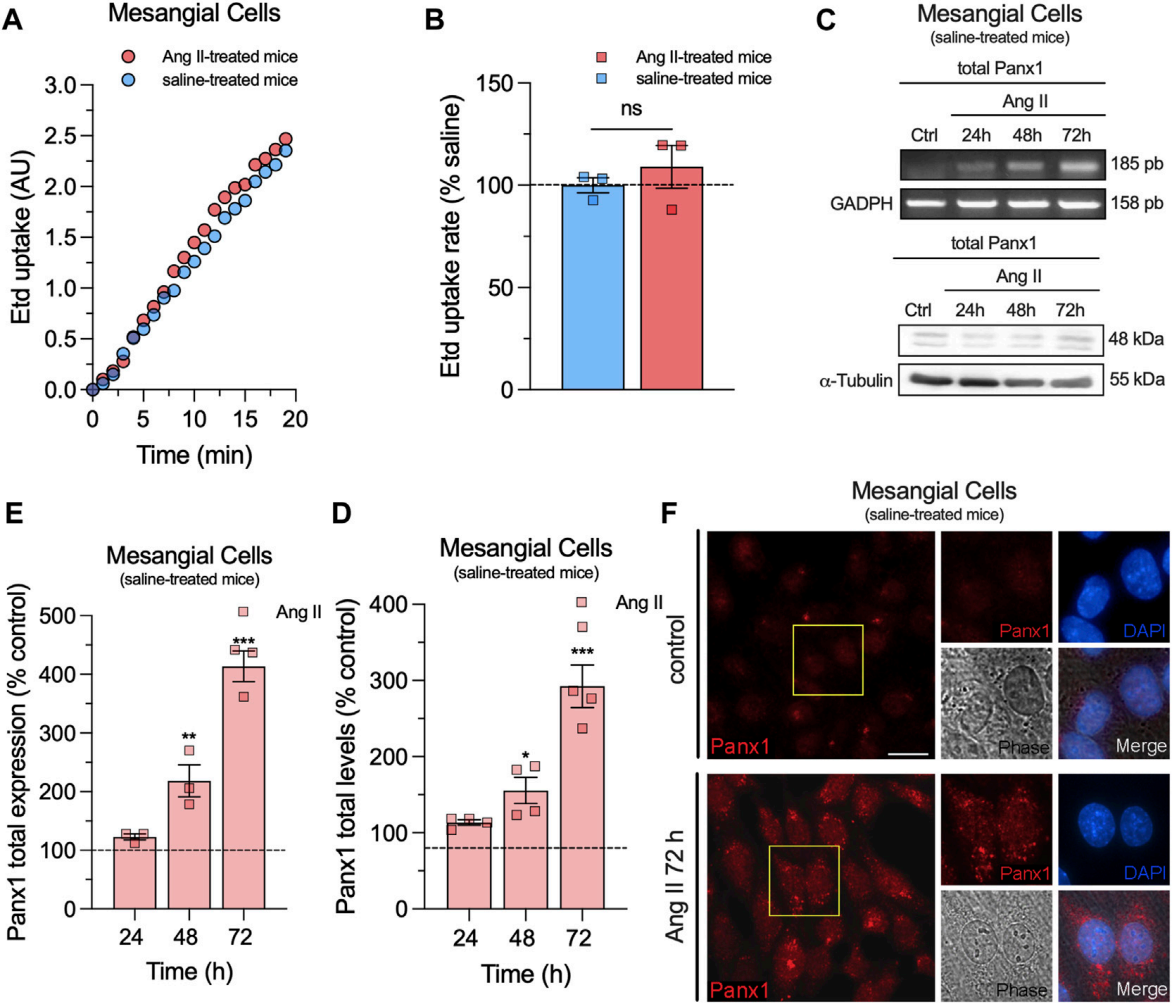
*In vivo* Ang II infusion does not impact Etd uptake, whereas Ang II *in vitro* enhances Panx1 expression in mesangial cells. **(A)** Time-lapse measurements of Etd uptake by cultured mesangial cells from saline-treated mice (blue circles) or mice infused with Ang II for 6 weeks (red circles). **(B)** Averaged Etd uptake rate normalized with saline condition (dashed line) by cultured mesangial cells from saline-treated mice (blue bars) or mice infused with Ang II for 6 weeks (red bars). Data were obtained from at least three independent experiments with four or more repeats each one (≥30 cells analyzed for each repeat). **(C)** Representative experiments of expression and protein levels of Panx1 by cultured mesangial cells from saline-treated mice under control conditions or treated with 1 μM Ang II for 24, 48 or 72 h. Levels of each analyzed band were normalized according to the levels of GADPH, and α-tubulin detected in each lane. **(D, E)** Quantification of expression **(D)** and total protein levels **(E)** of Panx1 normalized with the control condition (dashed line) by cultured mesangial cells from saline-treated mice stimulated with 1 μM Ang II for 24, 48 or 72 h. Each bar represents the mean value ± SEM of ≥4 independent experiments with four replicates each. Statistical significance, ***p < 0.001, **p < 0.01, *p < 0.05; Ang II vs control conditions (one-way ANOVA followed by Tukey’s *post hoc* test). **(F)** Representative immunofluorescence images depicting Panx1 (red) and DAPI (blue) staining by mesangial cells from saline-treated mice under control conditions or after treatment with 1 μM Ang II for 72 h. Calibration bar = 30 μm.

**FIGURE 3:**
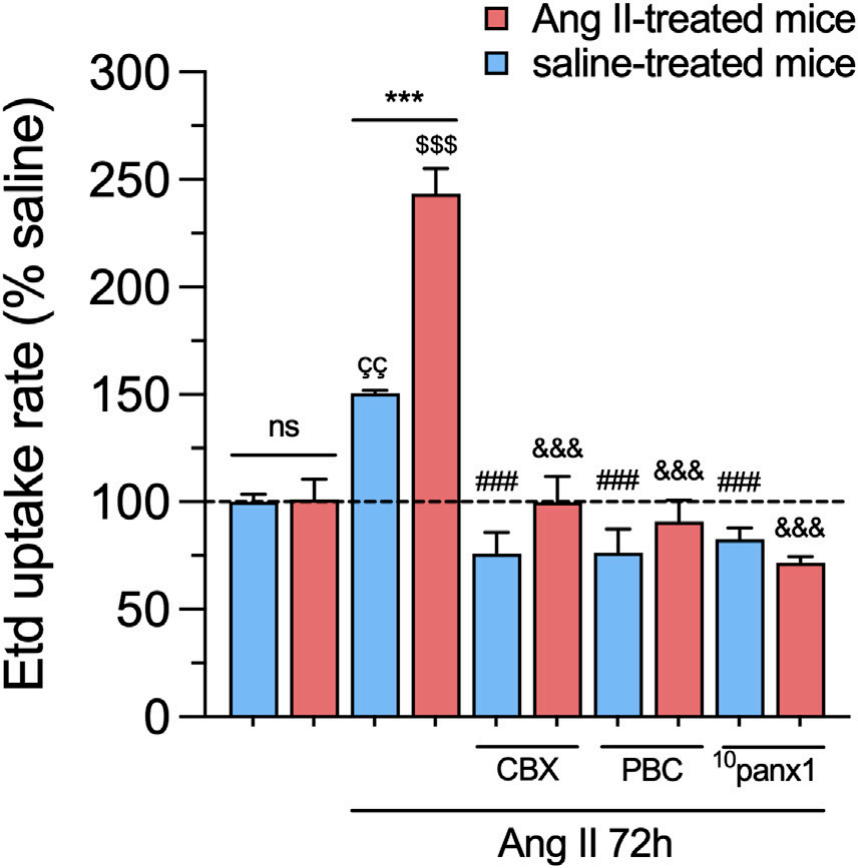
*In vivo* Ang II infusion sensitizes mesangial cells to exhibit increased Panx1 channel activity following a subsequent *in vitro* Ang II exposure. Averaged Etd uptake rate normalized with saline condition (dashed line) by mesangial cells from saline-treated mice (blue bars) or six-week-treated Ang II mice (red bars) under control conditions or stimulated with 1 μM of Ang II for 72 h alone or in combination with the following blockers: 10 µM carbenoxolone (CBX), 500 µM Probenecid (PBC), 50 µM ^10^panx1. Each bar represents the mean value ± SEM of ≥4 independent experiments with four replicates each. Statistical significance, ***p < 0.001; Ang II from Ang II-treated mice compared to Ang II from saline-treated mice; ^ÇÇ^p < 0.01; Ang II from saline-treated mice compared to saline-treated mice; ^$$$^ *p* < 0.001; Ang II from Ang II-treated mice compared to Ang II-treated mice; ^###^ *p* < 0.001; effect of pharmacological agents compared to Ang II treatment from saline-treated mice; ^&&&^ *p* < 0.001; effect of pharmacological agents compared to Ang II treatment from Ang II-treated mice (one-way ANOVA followed by Tukey’s *post hoc* test).

To investigate whether *in vivo* Ang II infusion increases Panx1 channel activity in mesangial cells, we measured the uptake of Etd. This molecule crosses the plasma membrane in healthy cells through selective large-pore channels, including Panx1 channels (Orellana et al., 2012). Upon intercalation with base pairs of DNA and RNA, Etd becomes fluorescent, reflecting the activity of channels (Johnson et al., 2016). Interestingly, primary cultures of mesangial cells derived from animals treated with Ang II for 6 weeks did not exhibit significant differences in Etd uptake compared to mesangial cells from saline-treated mice (Figures 2A,B). Previous studies have shown that various pathological conditions can sensitize cells to exhibit Panx1 channel activity only when subjected to a subsequent pathological challenge (Orellana et al., 2014; Cibelli et al., 2022). With this in mind, we further evaluated whether *in vivo* Ang II infusion could sensitize mesangial cells on its response to a subsequent *in vitro* exposure to Ang II. For that purpose, we first examined whether *in vitro* Ang II exposure affects the expression and levels of Panx1 in mesangial cells derived from saline-treated mice. We observed that 1 µM Ang II induced a time-dependent increase in Panx1 expression and protein levels, with 72 h of treatment representing the period that elicited the maximum effect (Figures 2C–F). In this context, we opted for the treatment of 1 µM Ang II for 72 h in further experiments. Interestingly, this treatment led to a significant increase in Etd uptake in mesangial cells compared to control conditions (untreated cells) (Figure 3). Strikingly, this response was notably higher in cells derived from mice infused with Ang II for 6 weeks compared to those treated only with saline (Ang II: 3.5-fold vs saline: 1.5-fold) (Figure 3). Given that Panx1 channels represent one of the primary conduits for Etd influx in the mesangial cell line MES-13 (Gómez et al., 2018), we further explored the potential involvement of these channels in the Ang II-induced Etd uptake observed in our system. Remarkably, 10 μM CBX and 500 μM PBC, two well-known blockers of Panx1 channels (Crespo Yanguas et al., 2018; Caufriez et al., 2023), completely suppressed the Ang II-induced Etd uptake in mesangial cells derived from Ang II- or saline-treated mice (Figure 3). Supporting these data, a similar inhibitory effect was found with the mimetic peptide ^10^panx1 (50 µM), which specifically inhibit Panx1 channel activity by binding the first extracellular loop of Panx1 (Pelegrin and Surprenant, 2006; Caufriez et al., 2023). These findings highlight that *in vivo* Ang II infusion sensitizes mesangial cells to show increased Panx1 channel activity in response to a subsequent *in vitro* exposure to Ang II.

### 3.2 *In vivo* Ang II infusion sensitizes mesangial cells to release higher levels of ATP and display elevated ATP-dependent [Ca^2+^]_i_ dynamics linked to the activation of Panx1 channels

**FIGURE 4:**
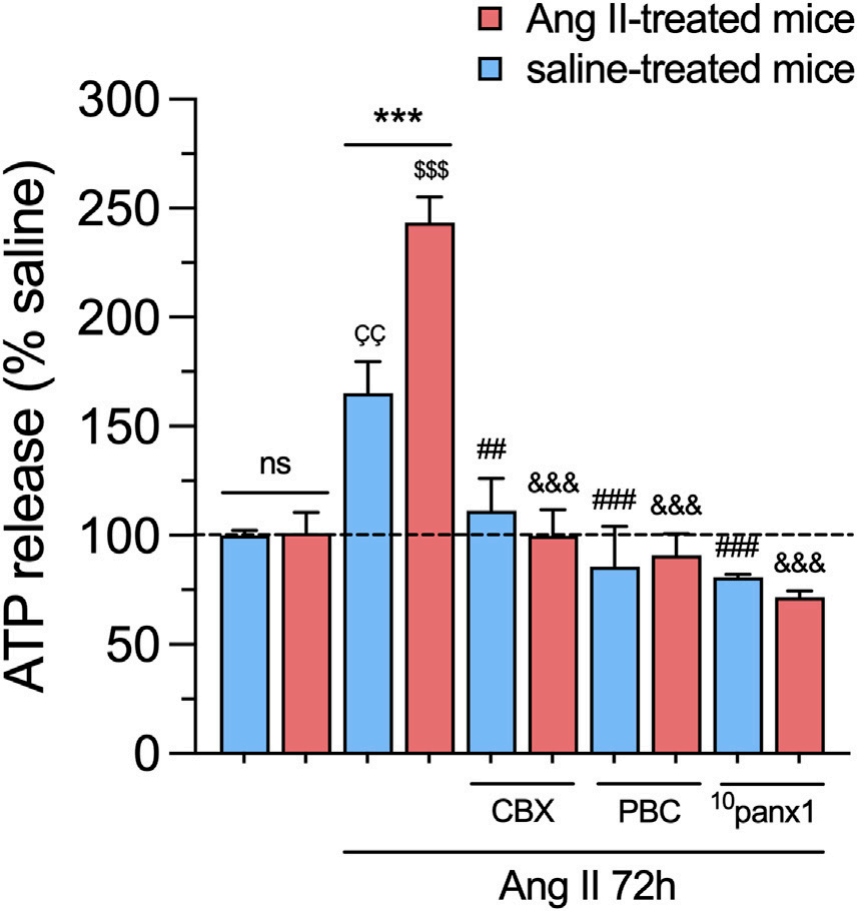
*In vivo* Ang II infusion sensitizes mesangial cells to exhibit increased Panx1 channel-dependent release of ATP following a subsequent *in vitro* Ang II exposure. Averaged data of ATP release normalized with saline condition (dashed line) by mesangial cells from saline-treated mice (blue bars) or six-week-treated Ang II mice (red bars) under control conditions or stimulated with 1 μM Ang II for 72 h alone or in combination with the following blockers: 10 µM carbenoxolone (CBX), 500 µM Probenecid (PBC) or 50 µM ^10^panx1. Each bar represents the mean value ± SEM of ≥4 independent experiments with four replicates each. Statistical significance, ***p < 0.001; Ang II from Ang II-treated mice compared to Ang II from saline-treated mice; ^ÇÇ^p < 0.01; Ang II from saline-treated mice compared to saline-treated mice; ^$$$^ *p* < 0.001; Ang II from Ang II-treated mice compared to Ang II-treated mice; ^##^ *p* < 0.01, ^###^ *p* < 0.001; effect of pharmacological agents compared to Ang II treatment from saline-treated mice; ^&&&^ *p* < 0.001; effect of pharmacological agents compared to Ang II treatment from Ang II-treated mice (one-way ANOVA followed by Tukey’s *post hoc* test).

Over the past decade, a body of research has demonstrated that the persistent activation of Panx1 channels contributes to the pathogenesis and progression of various diseases (Penuela et al., 2014). For instance, Panx1 channel activation can trigger the release of molecules, such as ATP, which, when secreted at high concentrations, may exert toxicity on neighboring cells (Abudara et al., 2018). In this context, we examined whether *in vivo* and/or *in vitro* treatment with Ang II could alter the release of ATP in primary mesangial cell cultures. As observed in experiments measuring Etd uptake, the baseline release of ATP remains unchanged in mesangial cells from mice treated with Ang II for 6 weeks compared to those infused only with saline (Figure 4). However, treatment with 1 µM Ang II for 72 h resulted in a substantial elevation in the release of ATP compared to untreated cells, which was comparatively higher in mesangial cells derived from mice infused with Ang II than saline (Figure 4). Notably, these responses were totally attenuated by blocking Panx1 channels with 10 µM CBX, 500 µM PBC, or 50 µM ^10^panx1 (Figure 4).

**FIGURE 5:**
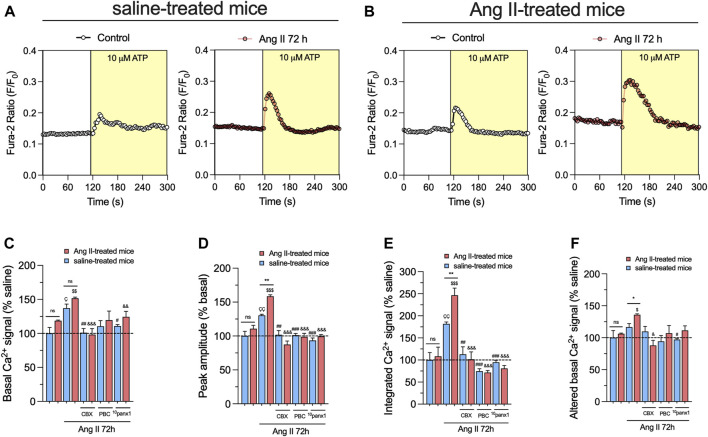
Panx1 channels contribute to the *in vivo* Ang II-induced sensitization of mesangial cells to exhibit increased basal and ATP-mediated [Ca^2+^]_i_ dynamics following a subsequent *in vitro* Ang II exposure. **(A, B)** Representative plots of relative changes in Ca^2+^ signals over time induced by 10 µM ATP (light yellow background) by mesangial cells from saline-treated mice **(A)** or six-week-treated Ang II mice **(B)** under control conditions (white circles) or stimulated with 1 μM Ang II for 72 h (orange circles). **(C–F)** Averaged data normalized with saline condition (dashed line) of basal Ca^2+^ signal **(C)**, ATP-induced peak amplitude normalized to basal FURA-2 ratio **(D)**, integrated ATP-induced FURA-2 ratio response **(E)** and altered basal FURA-2 ratio **(F)** by mesangial cells from saline-treated mice (blue bars) or six-week-treated Ang II mice (red bars) under control conditions or stimulated with 1 μM Ang II for 72 h alone or in combination with the following blockers: 10 µM carbenoxolone (CBX), 500 µM Probenecid (PBC) or 50 µM ^10^panx1. Each bar represents the mean value ± SEM of ≥4 independent experiments with four replicates each. Statistical significance, *p < 0.05, **p < 0.01; Ang II from Ang II-treated mice compared to Ang II from saline-treated mice; ^Ç^p < 0.05, ^ÇÇ^p < 0.01; Ang II from saline-treated mice compared to saline-treated mice; ^$^ *p* < 0.05, ^$$^ *p* < 0.01, ^$$$^ *p* < 0.001; Ang II from Ang II-treated mice compared to Ang II-treated mice; ^#^ *p* < 0.05, ^##^ *p* < 0.01, ^###^ *p* < 0.001; effect of pharmacological agents compared to Ang II treatment from saline-treated mice; ^&^ *p* < 0.05, ^&&^ *p* < 0.01, ^&&&^ *p* < 0.001; effect of pharmacological agents compared to Ang II treatment from Ang II-treated mice (one-way ANOVA followed by Tukey’s *post hoc* test).

The increase in [Ca^2+^]_i_ has been linked with heightened Panx1 channel activity in various cell types (Zorzi et al., 2017; Grimmer et al., 2022; Jaque-Fernandez et al., 2023). This facilitates the release of ATP and further engagement of purinergic receptors, perpetuating the sustained opening of Panx1 channels (Sáez et al., 2013; Garré et al., 2016). Accordingly, we examined whether *in vivo* and/or *in vitro* treatment with Ang II could modulate basal [Ca^2+^]_i_ levels in primary mesangial cell cultures. FURA-2 ratio (340/380) time-lapse recordings showed that basal [Ca^2+^]_i_ in mesangial cells from Ang II-or saline-treated mice remained unchanged (Figures 5A–C). Nevertheless, upon treatment with 1 µM Ang II for 72 h, mesangial cells showed a rise in basal [Ca^2+^]_i_ levels compared to untreated cells (Figures 5A–C). As occurred with the release of ATP, the above effect was more robust in mesangial cells derived from mice treated with Ang II for 6 weeks than those infused with saline (Figures 5A–C). Because we found in our system that Ang II triggers the release of ATP via Panx1 channels, we investigated the effect of Ang II on [Ca^2+^]_i_ responses elicited by this paracrine messenger. As previously reported (Gutierrez et al., 1999), acute stimulation with 10 µM ATP in mesangial cells induced a rapid peak in [Ca^2+^]_i_ that rapidly returned to baseline levels (Figures 5A,B). Relevantly, *in vitro* treatment with Ang II for 72 h augmented the ATP-induced amplitude of the peak and the integrated [Ca^2+^]_i_ signal (Figures 5D,E). Notably, these responses were more prominent in mesangial cells derived from mice treated with Ang II for 6 weeks than those infused with saline (Figures 5D,E). Of note, the remaining difference between the final and initial basal [Ca^2+^]_i_ signal triggered by ATP was elevated by *in vitro* Ang II treatment only in mesangial cells from Ang II-treated mice (Figure 5F).

Mounting evidence indicates that Panx1 channels indirectly elevate [Ca^2+^]_i_ via the ATP-dependent activation of purinergic receptors (Pinheiro et al., 2013b; Murali and Nurse, 2016; Murali et al., 2017). Consistent with this notion, we observed that inhibition of Panx1 channels with 10 µM CBX, 500 µM PBC, or 50 µM ^10^panx1 strongly diminished Ang II-induced increase in both [Ca^2+^]_i_ basal levels and ATP-mediated [Ca^2+^]_i_ signal amplitude (Figures 5A–D). Likewise, the blockade of Panx1 channels prominently abolished the augment in the integrated and remaining basal ATP-dependent [Ca^2+^]_i_ signal responses induced by the *in vitro* treatment with Ang II (Figures 5E,F). Overall, these data point out that the opening of Panx1 channels contributes to the *in vivo* Ang II-induced sensitization of mesangial cells in showing increased basal and ATP-induced [Ca^2+^]_i_ dynamics.

### 3.3 Panx1 channels contribute to lipid peroxidation, cytokine release, and cell death in mesangial cells from mice infused with Ang II

**FIGURE 6:**
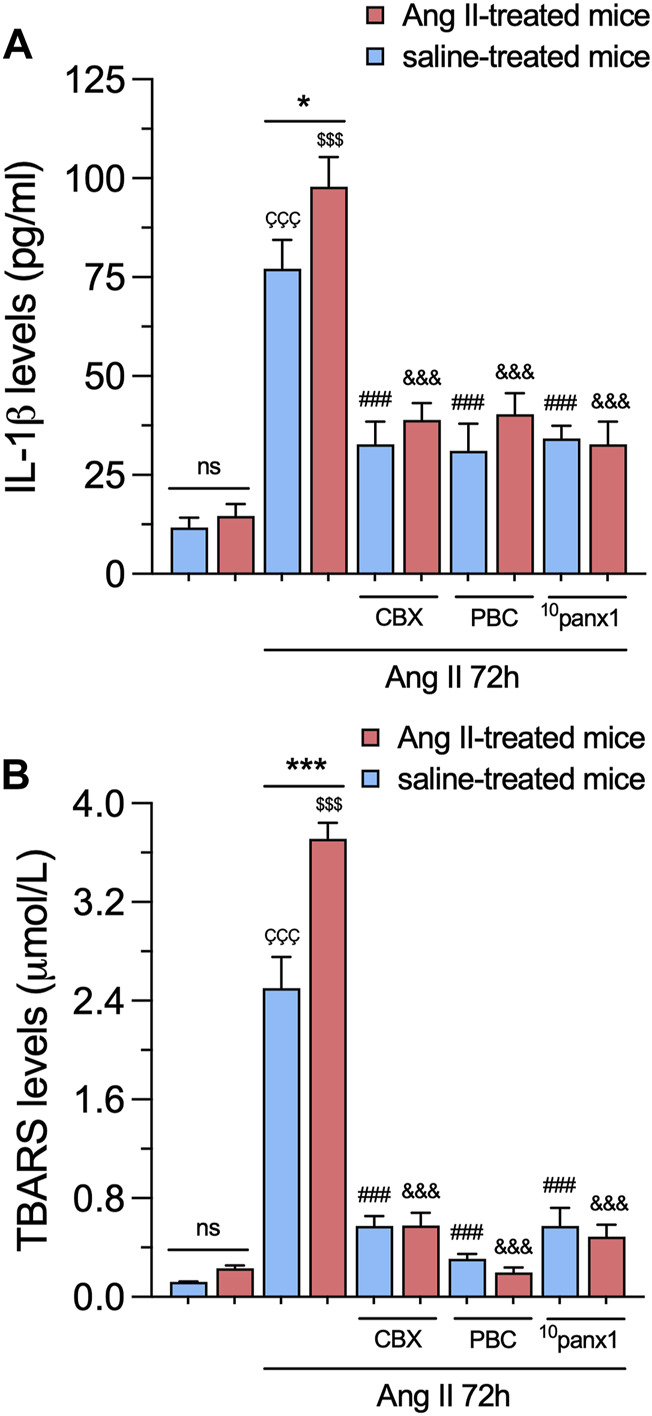
Panx1 channels contribute to the *in vivo* Ang II-induced sensitization of mesangial cells to exhibit increased IL-1β release and lipid peroxidation following a subsequent *in vitro* Ang II exposure. **(A, B)** Averaged data of IL-1β **(A)** or TBARS **(B)** levels by mesangial cells from saline-treated mice (blue bars) or six-week-treated Ang II mice (red bars) under control conditions or stimulated with 1 μM Ang II for 72 h alone or in combination with the following blockers: 10 µM carbenoxolone (CBX), 500 µM Probenecid (PBC) or 50 µM ^10^panx1. Each bar represents the mean value ± SEM of ≥4 independent experiments with four replicates each. Statistical significance, ***p < 0.001; Ang II from Ang II-treated mice compared to Ang II from saline-treated mice; ^ÇÇÇ^p < 0.001; Ang II from saline-treated mice compared to saline-treated mice; ^$$$^ *p* < 0.001; Ang II from Ang II-treated mice compared to Ang II-treated mice; ^###^ *p* < 0.001; effect of pharmacological agents compared to Ang II treatment from saline-treated mice; ^&&&^ *p* < 0.001; effect of pharmacological agents compared to Ang II treatment from Ang II-treated mice (one-way ANOVA followed by Tukey’s *post hoc* test).

In various renal pathologies, Ang II is recognized for its capacity to generate inflammatory mediators, including cytokines and oxygen radicals (Peng et al., 2008; Gómez et al., 2018). In this scenario, we scrutinized whether *in vitro* and/or *in vivo* treatment with Ang II affects the production of pro-inflammatory cytokines and oxidative stress in mesangial cells. For that purpose, we measured the production of IL-1β by ELISA and the generation of thiobarbituric acid reactive substances (TBARS), which are formed as a byproduct of lipid peroxidation. We found that *in vitro* treatment with 1 µM Ang II for 72 h elicited a marked increase in the extracellular levels of IL-1β and TBARS when contrasted with untreated cells (Figures 6A,B). These effects were significantly greater in mesangial cells from Ang II-treated mice than those infused with saline (Figures 6A,B). No changes in the production of IL-1β or TBARS were detected between control mesangial cells from mice infused with Ang II or saline (Figures 6A,B). Remarkably, the Ang II-induced augment in IL-1β or TBARS production was strongly mitigated upon blockade of Panx1 channels with 10 µM CBX, 500 µM PBC, or 50 µM ^10^panx1 (Figures 6A,B).

**FIGURE 7:**
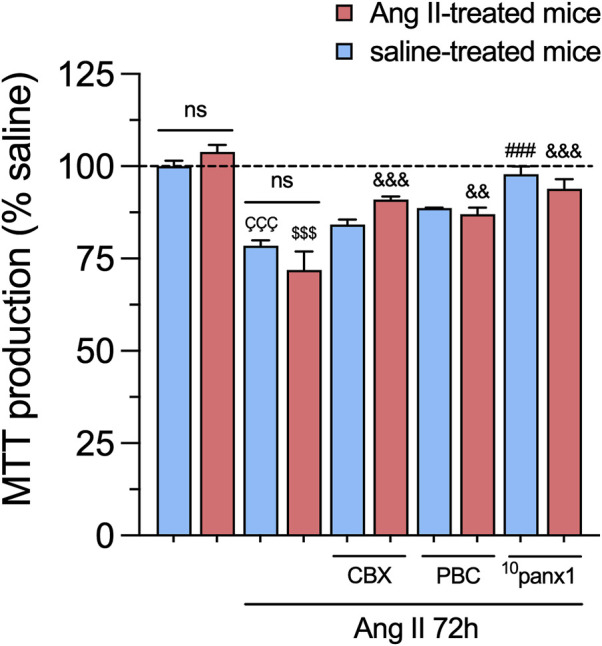
Panx1 channels contribute to the *in vitro* Ang II-induced decrease in cell viability in mesangial cells. Graphs showing the measurement of cell viability (MTT production) normalized with saline condition (dashed line) by mesangial cells from saline-treated mice (blue bars) or six-week-treated Ang II mice (red bars) under control conditions or stimulated with 1 μM Ang II for 72 h alone or in combination with the following blockers: 10 µM carbenoxolone (CBX), 500 µM Probenecid (PBC) or 50 µM ^10^panx1. Each bar represents the mean value ± SEM of ≥4 independent experiments with four replicates each. Statistical significance, ^ÇÇÇ^p < 0.001; Ang II from saline-treated mice compared to saline-treated mice; ^$$$^ *p* < 0.001; Ang II from Ang II-treated mice compared to Ang II-treated mice; ^###^ *p* < 0.001; effect of pharmacological agents compared to Ang II treatment from saline-treated mice; ^&&^ *p* < 0.01, ^&&&^ *p* < 0.001; effect of pharmacological agents compared to Ang II treatment from Ang II-treated mice (one-way ANOVA followed by Tukey’s *post hoc* test).

We further examined the potential impact of *in vitro* and/or *in vivo* treatment with Ang II on mesangial cell survival. To assess this, we measured cell viability by quantifying the reduction of MTT to formazan, a process directly correlated with the number of metabolically active cells in the culture. Mesangial cells stimulated with 1 µM Ang II for 72 h exhibited a substantial reduction in cell viability compared to untreated cells (Figure 7). Interestingly, no changes in MTT production were observed in mesangial cells from mice infused with either Ang II or saline, regardless of whether they were treated with Ang II *in vitro* or not (Figure 7). Relevantly, 50 µM ^10^panx1 completely prevented the Ang II-induced increase in MTT production in mesangial cells, whereas 10 μM CBX or 500 µM PBC only were effective in cells derived from Ang II-treated mice (Figure 7). These findings highlight the pivotal role of Panx1 channels in the Ang II-induced cell death and production of pro-inflammatory cytokines and lipid peroxidation in mesangial cells.

## 4 Discussion

This study marks the first evidence showing that *in vivo* Ang II infusion sensitizes cultured mesangial cells, leading to increased activity of Panx1 channels upon a subsequent *in vitro* stimulation with Ang II. Interestingly, Panx1 channel activation is critical in the sensitization induced by *in vivo* infusion with Ang II in various aspects of mesangial cell function. This includes the release of ATP, basal and ATP-induced [Ca^2+^]_i_ dynamics, lipid peroxidation and cell survival.

By conducting time-lapse recordings, we observed an increase in Etd uptake in mesangial cells following *in vitro* stimulation with Ang II. This response was notably potentiated when mesangial cells were derived from Ang II-induced hypertensive mice. The implication of Panx1 channels in Ang II-induced Etd uptake in mesangial cells was verified using well-known blockers (CBX and PBC) and a selective mimetic peptide (^10^panx1) to antagonize these channels. These results align with previous studies demonstrating that Ang II triggers the activation of Panx1 channels in the mesangial cell line MES-13 (Gómez et al., 2018) and rat carotid body type II cells (Murali et al., 2014).

Extracellular nucleotides, especially ATP, participate in physiological and pathological processes by stimulating P2 purinergic receptors (P2XR and P2YR). These receptors significantly impact renal vascular resistance, autoregulation, and tubular transport function (Nishiyama and Navar, 2002; Guan and Inscho, 2011; Menzies et al., 2017). Moreover, Ang II is known for inducing the release of ATP (Katsuragi et al., 1996), and the release of this messenger through Panx1 channels could exacerbate cell damage by disrupting [Ca^2+^]_i_ dynamics and [Ca^2+^]_i_ homeostasis (Pinheiro et al., 2013b; 2013a; Muñoz et al., 2015; Jaque-Fernandez et al., 2023). Here, we discovered that the activation of Panx1 channels was essential for the Ang II-induced release of ATP in mesangial cells. These results harmonize with previous studies emphasizing ATP release via Panx1 channels in cells subjected to different pathological conditions (Pelegrin and Surprenant, 2006; Suadicani et al., 2012; Zhang et al., 2012; Pinheiro et al., 2013b; Dahl et al., 2013; Krick et al., 2016). Cellular responses to ATP, including P2YR-dependent release of Ca^2+^ from internal stores and extracellular Ca^2+^ influx via P2XRs, are characteristic (Pelegrin and Surprenant, 2006; Suadicani et al., 2012; Zhang et al., 2012; Pinheiro et al., 2013b; Dahl et al., 2013; Krick et al., 2016). Mesangial cells stimulated with Ang II *in vitro* showed increased basal levels of [Ca^2+^]_i_, an effect potentiated by the *in vivo* infusion with Ang II. Similar sensitizing responses were found in several enhanced ATP-induced [Ca^2+^]_i_ responses, including signal amplitude, the integrated area under the curve, and sustained signal. CBX, PBC or ^10^panx1 totally prevented these responses, indicating that the Ang II-induced augment on ATP-induced [Ca^2+^]_i_ dynamics relies on the activation of Panx1 channels in mesangial cells.

The activation of AT1 receptors by Ang II is crucial for the progressive deterioration of glomerular function, contributing to the inflammatory and oxidative damage in renal diseases (Rupérez et al., 2005; Kolavennu et al., 2008). Consistent with this, we observed an elevated production of both TBARs and IL-1β in Ang II-stimulated mesangial cells. The latter response was enhanced in cells isolated from mice infused with Ang II, whereas blockade of Panx1 channels completely suppressed it. These data coincide with previous studies showing that Panx1 channel activity cooperates to produce free radicals, lipid peroxidation, and inflammation during kidney injury (Huang et al., 2020; Liu et al., 2022; Yin et al., 2022). Intriguingly, although *in vitro* stimulation with Ang II reduced the viability of mesangial cells due to the activation of Panx1 channels, no sensitization was detected by the *in vivo* infusion with Ang II. A possible explanation for this finding is that more extended periods of exposure to *in vitro* Ang II are required to observe the sensitizing effect of *in vivo* Ang II infusion on cell viability.

What mechanism does Ang II employ to enhance Panx1 channel activity, and how this impact mesangial cell pathophysiology? Ang II contracts mesangial cells by rapidly augmenting [Ca^2+^]_i_ upon binding to AT1 receptors (Feng et al., 2006; Qiu and Ji, 2014). The latter is crucial for the stretch reflex within the glomerulus, compensating the rise in filtration pressure with a reduction in capillary surface area (Stockand and Sansom, 1998). The Ang II-mediated augment in [Ca^2+^]_i_ activates stretch non-selective cation channels and Cl^−^ efflux, leading to plasma membrane depolarization (Craelius et al., 1989; 1993; Kremer et al., 1989; 1992; Matsunaga et al., 1991; Kleta et al., 1995). Subsequently, the altered membrane potential activates voltage-gated Ca^2+^ channels and receptor-operated Ca^2+^ channels, allowing Ca^2+^ influx and further activating depolarizing conductances (Ma et al., 2005). Interestingly, Panx1 channels typically mediate voltage-dependent, outwardly rectifying whole-cell currents (Bruzzone et al., 2003; Romanov et al., 2012; Sandilos et al., 2012; Jackson et al., 2014) and are activated by pressure or mechanical stretch (Bao et al., 2004). With this in mind, it’s conceivable that the initial depolarization or stretching triggered by Ang II could swiftly activate Panx1 channels in mesangial cells. However, given that in our system, the shortest treatment time with Ang II was 1 h, further studies are needed to verify if this hormone can rapidly modulate the activity of Panx1 channels. Alternatively, it is also possible that Panx1 channels contribute to the initial steps of mesangial contraction, such as depolarization and Ca^2+^ influx. Indeed, the opening of Panx1 channels results in large inward currents that sustain anoxic depolarization in pyramidal neurons (Weilinger et al., 2012). In addition, Panx1 channel activation could elevate [Ca^2+^]_i_ by releasing ATP and stimulating purinergic receptors (Dahl, 2015). On the other hand, our PCR, western blot, and immunocytochemistry data indicate that long-lasting *in vitro* exposure to Ang II increases the expression and protein levels of Panx1 channels in mesangial cells. This likely suggests an elevated number of Panx1 channels susceptible to being activated on the cell surface. In addition to its translational effects, Ang II could indirectly influence the activity of Panx1 channels. In this context, Ang II-mediated activation of Panx1 channels could occur due to a rise in [Ca^2+^]_i_. Supporting the latter idea, Murali and colleagues reported that Ang II augments Panx1 channel currents through AT1 receptor signaling in rat carotid body type II cells (Murali et al., 2014). The latter occurred primarily via the G-protein-coupled phosphatidylinositol–IP_3_ pathway. This evidence is consistent with the fact that the rise in [Ca^2+^]_i_ significantly enhances Panx1 channel activity (Locovei et al., 2006; López et al., 2021). Ang II-induced increase in [Ca^2+^]_i_ could lead to the subsequent Panx1-dependent release of ATP into the extracellular space (Dahl et al., 2013; Gómez et al., 2018), which may propagate the signaling effects of Ang II to neighboring cells, resulting in [Ca^2+^]_i_ responses that can impair mesangial cell function and survival. In fact, [Ca^2+^]_i_ handling defects could result in the generation of free radicals, lipid peroxidation, and damage to the plasma membrane (Abudara et al., 2018). Importantly, as Panx1 channel activation in the companion of P2XR/P2YR stimulation augments [Ca^2+^]_i_ (Pelegrin and Surprenant, 2006; Suadicani et al., 2012; Zhang et al., 2012; Pinheiro et al., 2013b; Dahl et al., 2013; Krick et al., 2016), they could contribute to perpetuating the propagation of ATP-mediated signaling. Indeed, high extracellular ATP levels are known to contribute to mesangial cell transformation and promote renal injury in Ang II-dependent hypertension (Vonend et al., 2004; Graciano et al., 2008). Moreover, previous evidence indicates that extracellular ATP induces apoptosis and necrosis in cultured mesangial cells through purinergic receptors (Schulze-Lohoff et al., 1998). These events may coincide with Panx1 channel-dependent cytokine release, including IL-1β, as demonstrated in other cell types (Pelegrin and Surprenant, 2006; Díaz et al., 2019).

Are there other potential channels responsible for ATP release that could co-regulate the opening of Panx1 channels upon Ang II stimulation? One possible candidate is the connexin-43 (Cx43) hemichannel. Like Panx1 channels, these channels facilitate communication between the cytoplasm and the extracellular environment, allowing the release of various molecules, including ATP. In hypertensive nephropathy, the elevated levels of Cx43 observed in the glomerulus and renal cortex emphasize its involvement in kidney injury (Gómez et al., 2018; Lucero et al., 2022b). Significantly, the ablation of Cx43 demonstrates a potential therapeutic avenue, improving renal function in chronic kidney disease induced by hypertension (Haefliger et al., 2006; Alonso et al., 2010; Abed et al., 2014; Yang et al., 2014; Lucero et al., 2022a). In the context of this study, it is noteworthy that in our previous work using the mesangial cell line MES-13, we demonstrated that Ang II not only increases the activity of Panx1 channels but also activates Cx43 hemichannels (Gómez et al., 2018). This activation leads to the release of IL-1β and TNF-α. More importantly, subsequent studies unveiled that both cytokines augment the activity of Cx43 hemichannels in primary cultures of mesangial cells (Lucero et al., 2022a). Overall, this evidence suggests that Ang II may induce a feed-forward mechanism of activation between Cx43 hemichannels and Panx1 channels. Future research is needed to determine whether the combined activation of both channels in mesangial cells contributes to kidney injury during hypertension.

Hypertension is one of the most prevalent risk factors for various health issues, including coronary artery disease, stroke, heart failure, peripheral arterial disease, vision loss, dementia, and CKD (Sequeira-Lopez and Gomez, 2021). The current study demonstrates that Panx1 channels could be crucial in orchestrating Ang II-induced adverse effects on mesangial cell function and survival. Additional investigation is needed to ascertain whether the opening of Panx1 channels in mesangial cells occurs during hypertension and its implications for the pathogenesis and progression of CKD. This research collectively contributes to advancing our understanding of cellular responses, signaling pathways, and regulatory mechanisms, with potential implications for renal function, blood pressure regulation, and vascular disorders. Further exploration in these areas promises to uncover novel therapeutic targets and deepen our knowledge of physiological processes. Our hypothesis posits that the activation of Panx1 channels, likely by the conventional RAS axis leads to elevated extracellular ATP levels, reaching toxic levels in the glomerulus. The latter could contribute to glomerular inflammation and fibrosis, thereby causing renal injury. Consequently, understanding the impact of Panx1 channel activity on mesangial cell signaling represents a promising avenue for developing therapeutic strategies addressing CKD.

## Data Availability

The original contributions presented in the study are included in the article/Supplementary material, further inquiries can be directed to the corresponding authors.
